# Genomic Consequences of Ecological Speciation in *Astyanax* Cavefish

**DOI:** 10.1371/journal.pone.0079903

**Published:** 2013-11-19

**Authors:** Richard Borowsky, Dana Cohen

**Affiliations:** Department of Biology, New York University, New York, United States of America; University of Calgary, Canada

## Abstract

The cave environment is consistently radically different than the surface environment because it lacks light, and animals adapting to cave life are subject to strong selective forces much different than those experienced by their ancestors who evolved in the presence of light. As such, their divergence from surface ancestors and eventual speciation is likely to be driven by the shift in ecology. We report here that hybrids between cave and surface *Astyanax mexicanus* fishes produce offspring with allelic frequencies that differ significantly from Mendelian expectations both for transmission ratios and for independent assortment of unlinked markers. Comparison of allelic content of DNA from fin clips and sperm pools show that the transmission ratio distortion likely occurs during spermatogenesis. Departures from expectations of independent assortment are essentially epistatic phenomena generating linkage disequilibrium. A novel analysis of the epistatic interactions reveals an apparent network of interactions among genes known or suspected to be involved in cave adaptation, implying that the epistasis arose as a “by product” of the divergence due to cave adaptation.

## Introduction

Divergent selection between dissimilar habitats can drive phenotypic differentiation of separate populations, with speciation an eventual result if the genetic changes lead to reproductive isolation. Speciation driven in this way is known as “ecological speciation” and is believed to come about when alleles selected in the new environment cause reproductive isolation as a pleiotropic “by product” of their selection [1], [2]. Attainment of speciation is thought to be facilitated by either 1) strong selection on as few as a single trait or 2) weaker selection on numerous traits. The first mechanism is termed “stronger selection” and the second is termed “multifarious selection” [3]. Although both can drive speciation, multifarious selection is hypothesized to be more effective than stronger selection because its genome wide basis leads to a more comprehensive correlated evolutionary response [3].

Cave adapted fishes and cave animals generally provide striking examples of ecologically driven phenotypic evolution affecting numerous diverse functions like vision, pigmentation, metabolism, chemo- and mechanosensory sensitivity, feeding behavior, general activity and sleep, among others [4]–[10]. There are so many changes that cave adaptation may involve both strong differential selection and multifarious traits [8], effectively facilitating reproductive isolation even in the presence of retarding factors such as continued gene flow.

The blind cavefish, *Astyanax mexicanus*, is well suited for studies of potential ecological speciation because it is interfertile with eyed surface relatives, which permits lab-based studies of the genetics of their differences. In nature, this ability to interbreed has the potential to counteract the divergence of the cave forms and it is known from population genetic analyses that there is significant bi-directional gene flow between surface and cave populations [11]. In spite of this gene flow, however, the two forms maintain their phenotypic differences in the field. This implies either strong continuing ecological selection or some degree of reproductive isolation, or both.

Our past mapping and QTL analyses of *Astyanax* cave/surface hybrids revealed that cave adaptation comes about through allelic substitutions at numerous genetic loci [6], [8], [9],[12]–[15], providing ample potential for correlated evolution of reproductive barriers. Early on in the mapping studies we observed that many loci exhibited significant patterns of transmission ratio distortion. The cause of these transmission biases were unknown, but implied considerable divergence and incompatibility of the cave and surface genomes, suggesting the cave and surface forms were already distinct species. In order to learn more about these incompatibilities, we present here a thorough reanalysis of our earlier mapping data, as well as new experimental work. We report below two distinct patterns of non-random allelic transmission by F_1_ hybrids to offspring: 1) transmission ratio distortion and 2) epistatic departures from independent assortment of alleles at unlinked loci. Both may serve as post-mating reproductive barriers by reducing reproductive fitness of hybrids. The deviations from expectations are genome-wide and appear to reflect selection on the male germline rather than on the female germline or during the zygotic stage.

## Results

**Figure 1:**
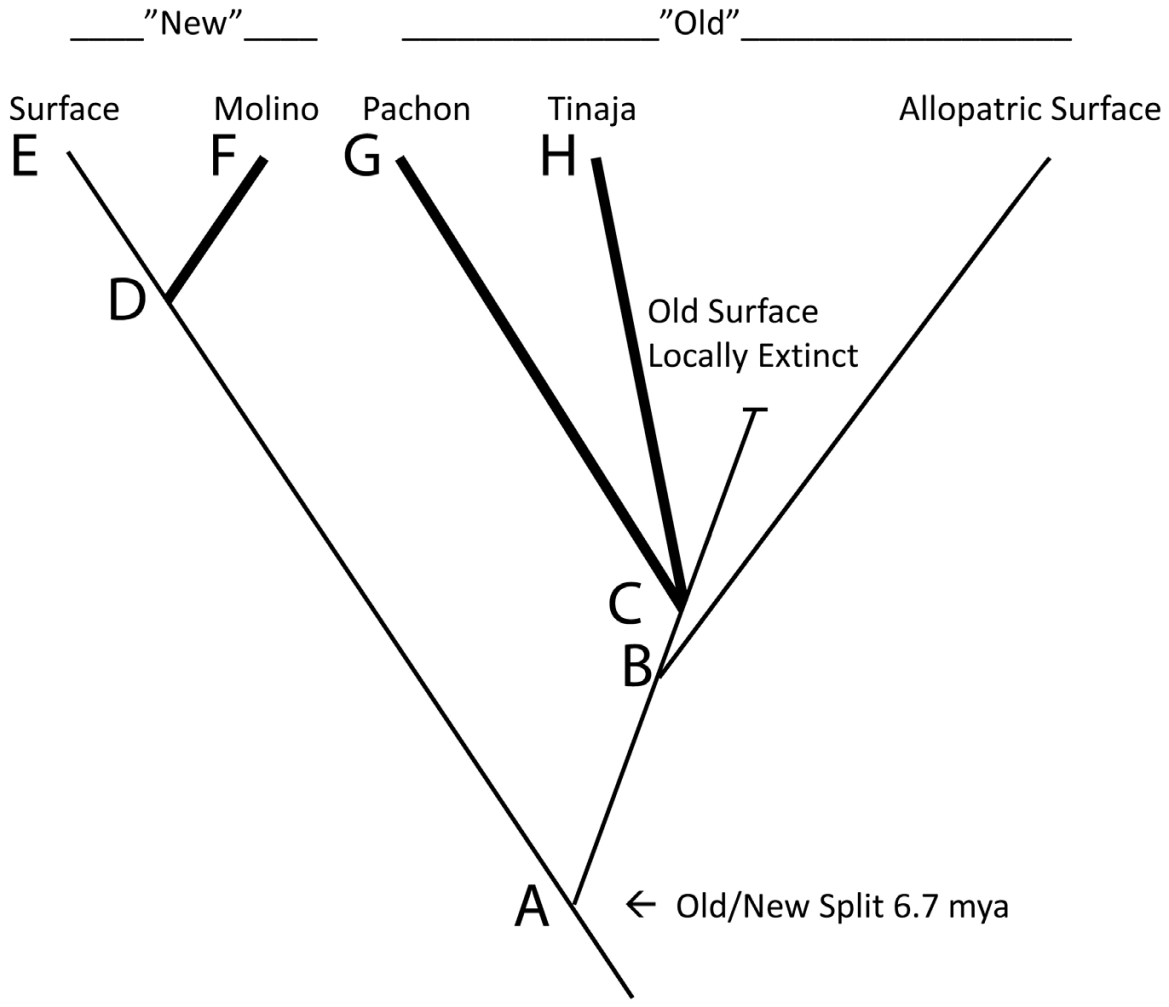
A simplified phylogeny of *Astyanax* showing the relationships of the populations discussed herein[17], [20]. The thick lines represent the subterranean phases of the cave lineages. Node A was estimated to be 6.7 mya [17]. No dating is available for the other nodes, except that node D is more recent than node C.

We analyzed patterns of allelic transmission ratio distortion in five previously reported F_2_ and backcross (BC) mapping crosses [6], [8], [9], [12]. Recent studies show that most of the *Astyanax* cave forms are of a lineage (“old stock”) that diverged from that of the present day surface fish (“new stock”) 6.7 mya [11], [16]–[19]. A simplified phylogeny is shown in Fig. 1. The hybrid crosses were between surface fish and cave fish from Pachón (F_2_ and BC), Tinaja (two F_2_ crosses) and Molino (BC), termed PSF2, PSBC, TSF2a, TSF2b, MSBC, respectively (Table S1). The Pachón and Tinaja cave populations are old stock; the Molino cave population, like the surface fish, is new stock.

### Transmission Ratio Distortion

For each locus we genotyped we counted the numbers of cave and surface alleles that were transmitted from the hybrid F_1_ to their respective F_2_ or backcross offspring. Each cross exhibited significant departures from Mendelian expectations although the departures were greater in the F_2_ than in the backcrosses. In the PSF2 cross, fully 28.7% (155 of 541) of the loci we mapped exhibited departures from a 1∶1 ratio that were nominally significant at the P = 0.05 level. The expected number of false-positives (i.e., Type I errors) would be 27 (0.05 * 541), implying that many of the remaining 128 were biologically significant outliers.

**Figure 2:**
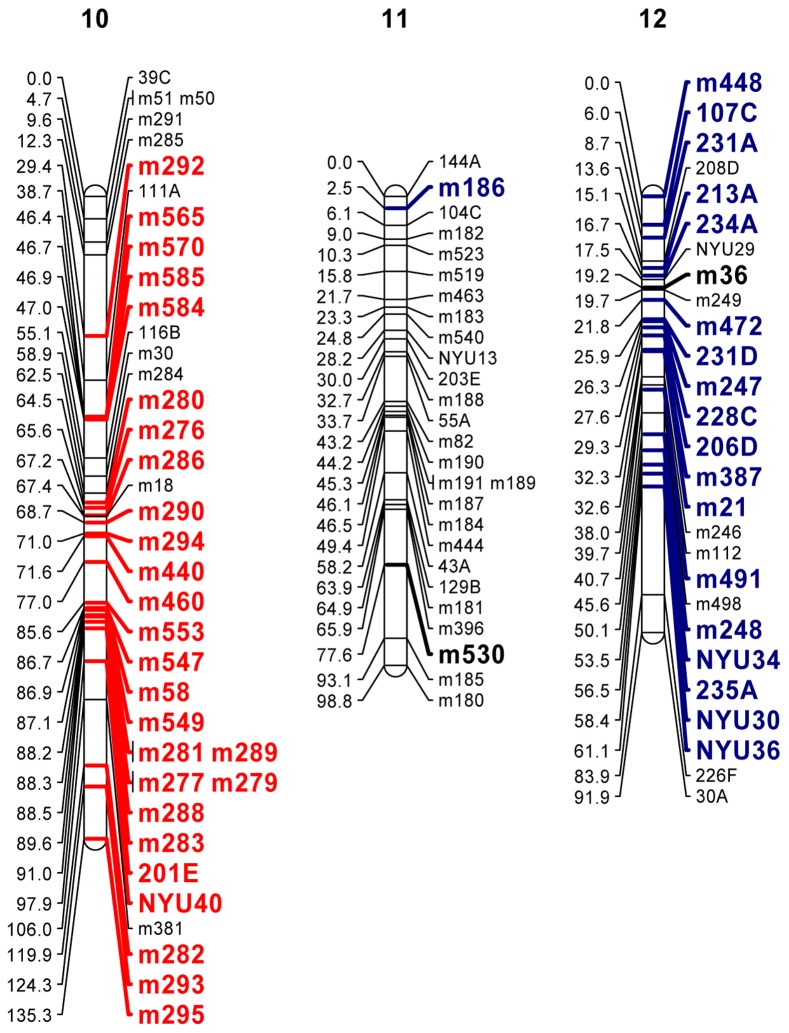
Three linkage groups (LGs) mapped in the PSF2 cross. Nearly all the loci on LG 10 exhibited preferential transmission of the surface alleles (red) while nearly all the loci on LG 12 exhibited preferential transmission of the Pachón cave alleles (blue). LG 11 exhibited little evidence of biased transmission. Full maps are found in Supporting Information. NB!: The linkage groups of *Astyanax* have not yet been standardized. All LG numbering is cross specific. LG numbers do not correspond between crosses.

Distorted loci were clustered in several regions of the genome. In some cases whole linkage groups were significantly biased towards transmission of the surface alleles, in other cases the bias favored the cave alleles. Figure 2 shows three linkage groups from the PSF2 cross as representative of the full range of biases. Ignoring isolated divergent loci and only counting clusters, there were 18 regions of significant bias found on 11 of the 26 mapped linkage groups in PSF2; these spanned 382 cM out of a total of 2584 cM, or 14.8% of the genome. (The full map for this cross is inFigure S1).

Looking at all loci that exhibited transmission biases at nominal significance levels of p = 0.001, we were able to identify 14 representative loci that were unlinked. For each of them we estimated the severity of zygotic selection that would have been required to produce the observed genotypic distributions from ideal 1∶2:1 starting distribution. We expressed this as the proportions surviving selection at each of the unlinked, independent loci. The cumulative product of these 14 values was 0.001285 (Table S2). By count, we established that maximum brood sizes are about 2,000 eggs. Thus, ova and zygotes are too few in number to be the basis for such a severely selected population.

The extensive genome-wide nature of the transmission bias instead suggests that it largely reflects events during spermatogenesis or through sperm competition. This implies that if the informative segregations are from a female parent, transmission bias should be greatly reduced or non-existent. To test this, we analyzed a Pachón/Surface BC progeny derived from a female F_1_ individual. As predicted, far fewer of the mapped loci exhibited significant bias (30 out of 431 = 7.0%, not significantly different from the expected false-positive rate of 5%, χ^2^ = 3.09, df = 1, p = NS), and the regions exhibiting bias covered only 2.6% of the genome (Figure S2). We observed the same in a backcross of a Molino/Surface hybrid female to a Molino male (MSBC), in which the regions of bias, as with PSBC, are fewer and far less extensive than those in the PSF2 cross (11.1% of the loci covering only 3.4% of the genome, Figure S3).

The Tinaja and Pachón cave populations were independently evolved from surface fish of the old lineage [20]. We reasoned that if the transmission bias exhibited in PSF2 reflected the genetic divergence between surface and cave fish and was a sperm associated phenomenon, it should also be manifest in the TSF2 crosses. As predicted, the TSF2a progeny exhibit transmission bias equivalent to that of the PSF2; 77 of 268 mapped loci (28.7%) exhibited bias and their clusters are found on 12 of the 24 linkage groups and span 11.6% of the genome (Figure S4). We also examined a second Tinaja/Surface F_2_, TSF2b. The two progenies are 25% related, having different sets of parents that were siblings. The TSF2b map covers less than 50% of the genome and its resolution is low because fewer individuals and loci were typed than in the other crosses, yet it shows the same pattern of numerous clusters of biased loci with 34 out of 131 mapped loci (26.0%) exhibiting significant bias and their clusters covering 4.6% of the genome (Figure S5).

To narrow down experimentally the stage at which transmission bias occurs, we compared allelic content of DNA obtained from the sperm of hybrid and non-hybrid males with that of control diploid DNA obtained from their fin clips. We used locus specific primers to amplify fragments from the pairs of DNA pools using published methods to tag the amplimers with fluorescent labels [9]. For each DNA sample and primer pair, ten independent amplifications were performed. Products were run on an ABI3730 to quantitate peaks, and the ratios of the peak heights for the two alleles were calculated (smallest divided by largest). The distribution of ratios for zygotic DNA (control) was taken to reflect its 1∶1 diploid allelic content and an associated measurement error. The control distributions were compared with those obtained from amplification of gametic DNA. A significant difference between the distributions of the ratios for the two DNA sources was taken as evidence that their allelic contents differed.

**Figure 3:**
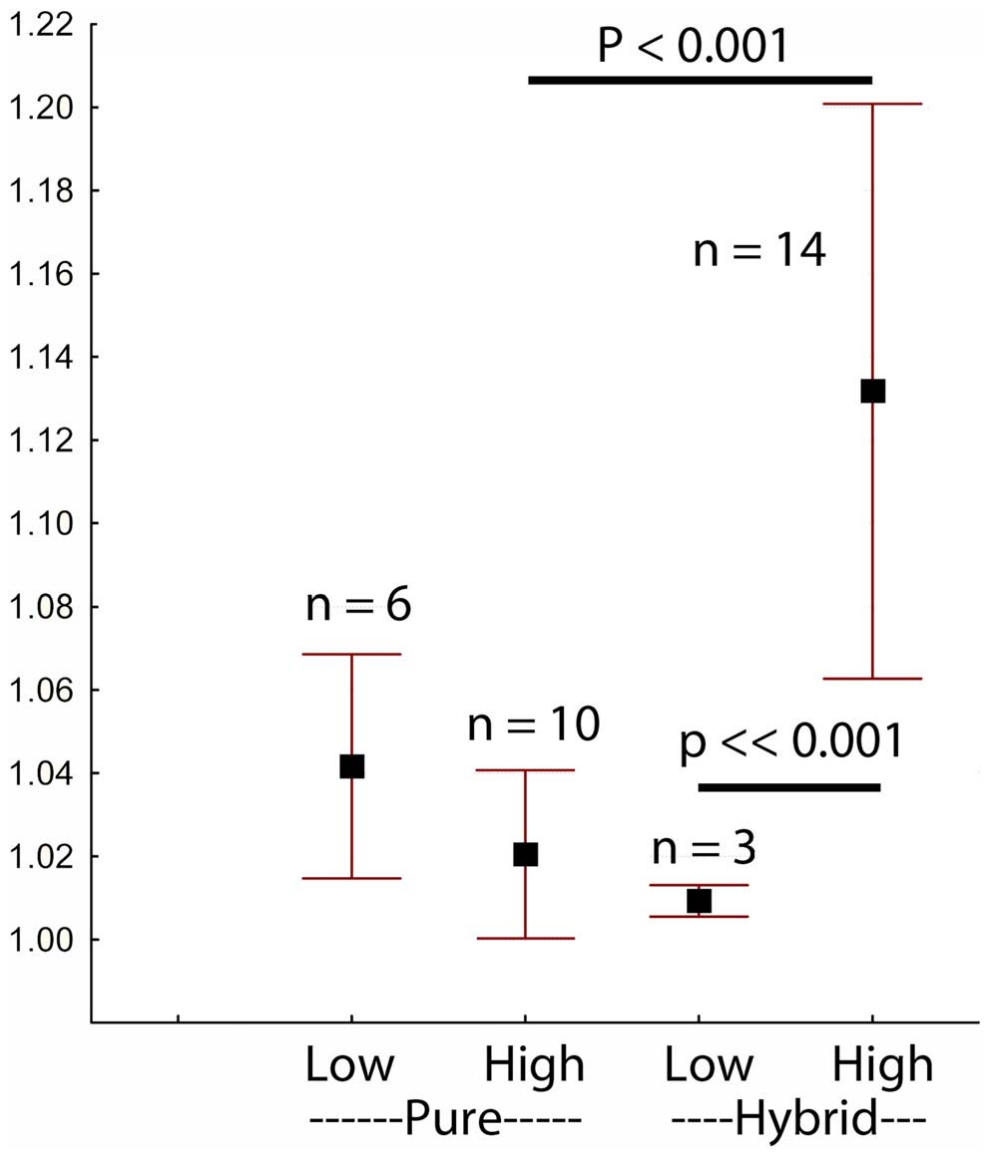
Allelic content of sperm DNA compared to that of zygotic (fin clip) control DNA. The ordinate is the ratio of the peak heights for the two alleles in gametic DNA divided by the corresponding ratio for control DNA, or its reciprocal, if less than 1.0. Four different classes of loci were tested: those that had exhibited low transmission bias in the pedigree analysis vs. those that had exhibited high transmission bias, further partitioned by whether the males tested were inter-lineage hybrids (hybrid) or non-hybrids/intra-lineage hybrids (pure). The hybrid/high class of loci was significantly more deviated from unity than either the hybrid/low or the pure/high classes.

Males that were F_1_ hybrids between lineages were compared with those that were non-hybrids or were F_1_ hybrids within a lineage (Table 1). Loci that exhibited extreme transmission distortion in the PSF2 cross were compared with those that had exhibited no evidence of transmission distortion. Thus, there were four groups for comparison. The prediction was that the group in which the males were inter-lineage hybrids and the loci tested had previously displayed significant distortion would exhibit significantly greater differences between gametic and zygotic DNA than seen in the other three groups. This prediction was borne out (Fig. 3). Thus, transmission bias occurs at a stage prior to full maturation of the sperm. Significantly, none of the males used to test this hypothesis were from a Pachón/Surface cross which was the origin of the male parent of PSF2. Thus, it is hybridization between lineages that causes the distortion; the specific populations appear irrelevant.

**Table 1:** Allelic biases in sperm from males that were non-hybrid or hybrid within a lineage versus from males that were hybrids across lineages.

			Non-hybrid	Hybrid Within:	Hybrid Across:		
	TRD	Locus	Various?	Asty136	Asty188	Asty41	Asty39
Cross				Pach/Tina	Yerb/Epi	Tina/Moli	Pach/Micos
High	2.42	NYU30	1.005	1.002			
	2.12	NYU36			1.205**	1.228**	1.066**
	1.77	27c	1.004	1.005	1.075*	1.194**	1.047
	1.76	24c			1.067*	1.088**	
	1.73	231c		1.001			
	1.73	229a	1.075			1.243**	1.443**
	1.58	107b				1.133**	
	1.39	208d	1.001, 1.006, 1.035*	1.002	1.014	1.004	1.043*
Low	1.26	216c	1.002, 1.035	1.037*		1.011	
	1.25	207b		1.036		1.008	
	1.08	219b	1.073**	1.067*		1.009	

Entries in the table are the ratios of peak heights for the two alleles segregating at the locus in DNA from sperm, standardized against the ratio of peak heights from zygotic DNA (fin clips from the same males). Larger numbers signify greater deviations from 1∶1 segregation in the sperm DNA. The TRD index was calculated from the observed allelic distributions in the PSF2 cross and is the ratio of the frequency of the more common allele divided by that of the less common allele. Not all loci were informative (heterozygous) in each male.

? Non-hybrid fish, one each from the surface population, and the Yerbaniz, Tinaja and Pachón cave populations. Abbreviations: Pach = Pachón cave, Yerb = Yerbaniz cave, Tina = Tinaja cave, Moli = Molino cave, Micos = Micos cave, Epi = epigean surface population. *p<0.05, **p<0.001. Asty136 is a hybrid F_2_ progeny within the old lineage. Asty188, Asty41, and Asty39 are hybrid F_2_ pedigrees across lineages.

We conclude the following for transmission ratio distortion: 1) the genomes of the old lineage cave fish from Pachón and Tinaja are mismatched with that of the new lineage surface fish to the point of causing major departures from Mendelian segregation in F_1_ hybrids. 2) Most of the transmission ratio distortion results from selection in the male germline rather than during oogenesis, because F_2_ progenies exhibit more extreme segregation distortion than backcross derived from hybrid females. 3) The transmission distortion in F_1_ males occurs prior to maturation of the sperm. 4) Because of the large number of selective deaths that would be needed to generate the pervasive segregation distortion, zygotic selection plays only a minor role, if any, in generating the biases.

### Epistatic Interactions among Loci

The hypothesis that the transmission ratio distortion reflects a genomic mismatch between the parental populations is essentially one of epistasis; to wit, that alleles at loci in each of the populations work harmoniously with one another but work less well with alleles from the other. We cannot yet identify the important loci for the transmission bias phenomenon, but we can examine specific pairings of loci to look for other evidences of epistasis. We focused on the Pachón F_2_ cross because it had been genotyped most extensively and with markers for genes that were candidates for cave phenotypes. Given the long separation of the old and new lineages and the genotypic remodeling accompanying adaptation to the cave environment, we hypothesized that the genome of the Pachón cave population would represent a newly derived coadapted gene complex. A simple test of this was to tabulate the frequencies of genotypic combinations over all pairs of unlinked loci in the F_2_, concentrating on the combinations of homozygotes. The null hypothesis is that the frequencies of homospecific genotypic combinations (AABB or aabb, lower case being cave allele) should equal those of heterospecific combinations (AAbb or aaBB) for any pairwise combination of unlinked loci. When the sum of AABB and aabb exceeded the sum of AAbb and aaBB we designated the pair as “Hom.” When the opposite was true we designated the pair as “Het.” To quantify the degree of inequality we calculated a χ^2^ value for the pair without Yates’ correction for continuity (expected value of χ^2^ = 1).

The results strongly suggested genome wide patterns of epistasis. The total number of Hom (49, 405) greatly exceeded that of Het (44, 068) (Table S3). For χ^2^ values exceeding 1.0, the discrepancy was even more striking (14, 854 vs. 9, 579). We binned the results by χ^2^ values (0 to 1, 1 to 2, etc. up to 13+); Hom exceeded Het in every bin (Table S3). While the difference in frequencies of the two combinations is striking and suggestive of coadaptation, it is difficult to assess its statistical significance because the pairwise comparisons are highly correlated through linkage. Also, there is no specific genetic hypothesis that anonymous markers can suggest. Therefore, we turned our attention to a set of 22 genes that had previously been mapped in the pedigree. These were not chosen at random, but had been selected for mapping because of their potential importance in cave adaptation. The genes included some known to be involved with eye development, pigmentation, apoptosis, etc. First, we compared the 3×3 matrices of genotypic combinations for all 215 pairwise combinations of genes that are unlinked and calculated χ^2^ values, each with four degrees of freedom. Since each individual comparison is an independent test of the hypothesis of independence of segregation, the overall significance was obtained by summing the χ^2^ and df values (Σχ^2^ = 981, Σdf = 864: p = 0.0025). Thus, we reject the hypothesis of genotypic independence; the loci, or others closely linked to them, are exhibiting epistatic interactions that generate linkage disequilibrium. In addition, these interactions were significantly skewed to Hom>Het (χ^2^ = 267, df = 215, p = 0.0091, summing χ^2^ values over all 215 pairwise combinations).

**Figure 4:**
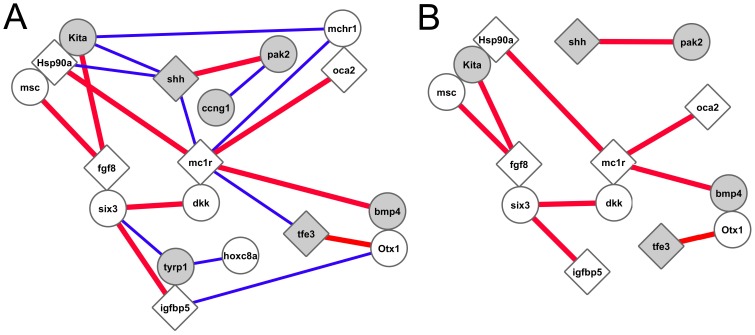
A network of genes (or closely linked loci) exhibiting significant epistatic interactions. Nodes are genes and edges denote epistatic interactions significant at p<0.05 (χ^2^>9.5, df = 4, analysis of a 3by 3 genotype matrix). Genes depicted are candidates for cave related phenotypes. The red edges signify interactions in which the ratio of Hom/Het was significantly greater than 1.0 (χ^2^>3.85, df = 1). Nodes in contact with one another signify genes that are linked.

Nineteen of the total 215 pairwise combinations involving 18 genes were individually significant at or below p<0.05 in the 3 by 3 matrix test. These putative interactions are depicted as a network in Figure 4a, where nodes represent genes and edges represent interactions. The diamond shaped nodes represent genes already known to be involved with cave adaptation [9], [14], [21]–[23]. The seven shaded nodes represent genes that displayed significant transmission ratio distortion. This number is not significantly different than the expected value of 5.2 based on the overall prevalence of markers exhibiting transmission bias. Approximately half of the 19 edges are false positives because the expected number of nominally significant edges is 10.25 based on the Type I error rate. Nevertheless, 19 significantly exceeds 10.25 (Yates corrected χ^2^ = 5.88, df = 1, p = 0.0153), so a subset of the interactions are biologically significant.

As indicated above, we used two different χ^2^ tests to detect epistasis. The first was based on the 3 by 3 matrix listing all nine two-locus genotypic frequencies and the second was based on the expectation of equality of Hom and Het. While related, the two tests are largely independent (r^2^ = 0.24), and thus the second test can provide additional information on the putative interactions identified by the first. We found that nine of the 19 pair-wise comparisons were significant at or below p<0.05 based on the relative values of Hom and Het, and are thus likely to be biologically significant. In all nine of these cases Hom exceeded Het (Binomial p = 0.004), an observation consistent with the hypothesis that the interactions arise from genetic incompatibilities between the two genomes. The nine interactions are flagged in red in Figure 4a. Figure 4b shows the network reduced by the removal of the ten edges not significant by both tests.

The network suggests the testable and important hypothesis that cave alleles at or near these loci comprise a coadapted gene complex. To test whether there was an overall consensus pattern in the two-way comparisons we standardized the proportional representation in each of the nine cells of a pairwise comparison by dividing observed numbers by expectations obtained from marginal totals. We then averaged over all 18 comparisons. This revealed a clear pattern: homospecific combinations were overrepresented (by 17%) and heterospecific combinations were underrepresented (by 16%), with all other combinations close to expected representation (Table 2A). The differences among the three classes were highly significant (overall differences tested by ANOVA: F_2,141_ = 19.5, P<10^−6^; pairwise t tests: least significant p value <10^−3^).

**Table 2:** Homospecific combinations of alleles (HOM = hhHH or kkKK) at pairs of unlinked loci are significantly more common than heterospecific combinations (HET = hhKK or kkHH).

2A				2b			
	HH	HK	KK		HH	HK	KK
hh	1.198	0.974	0.832	hh	1.275	0.923	0.843
hk	0.974	1.012	1.016	hk	0.956	1.046	0.974
kk	0.844	1.013	1.138	kk	0.836	0.974	1.204
	**HOM**	**Others**	**HET**		**HOM**	**Others**	**HET**
Means	1.168	0.998	0.838	Means	1.24	0.975	0.84
SEM	0.046	0.018	0.047	SEM	0.055	0.025	0.086

Other combinations have frequencies close to expected values. 2A: Averages of the 19 pairwise comparisons from Figure 4. 2B: Averages of the six mc1r pairwise comparisons from Figure 4.

Six of the 19 edges in Figure 4a involve mc1r, the most interconnected locus. The pattern for these six comparisons was even more striking, with homospecific combinations overrepresented by 24% (Table 2B). The strongest single interaction detected was between mc1r and oca2 (homospecific to heterospecific combinations = 70∶32; binomial p = 0.0002; Table 3). Both of these genes have known mutants in Pachón and several other cave populations which affect melanin pigmentation.

**Table 3:** Homospecific combinations of alleles at mc1r (H or K) and oca2 (h or k) are significantly more frequent than heterospecific combinations (binomial p = 0.0002).

Counts:				Proportional representation:			
	HH	HK	KK		HH	HK	KK
hh	33	53	22	hh	1.45	0.91	0.81
hk	55	136	58	hk	1.05	1.02	0.92
kk	10	60	37	kk	0.44	1.04	1.37
					HOM	Others	HET
				Means	1.41	1	0.69

It is possible that the strong epistatic interaction is due to linked genes and not between mc1r and oca2 directly, and merely reflects the genetic divergence of the two lineages (Fig. 1, Node A). To test this, we turned to the Tinaja/Surface cross (TSF2a), in which we had mapped oca2 using a closely linked SNP, and for two markers closely flanking mc1r (2–4 cM distant). Alleles for both oca2 and mc1r in the Tinaja population are fully functional. Thus, while the PSF2 cross represents lineage hybridization as well as all combinations of functional and nonfunctional oca2 and mc1r genotypes, the TSF2a cross represents only lineage hybridization. Both pairwise comparisons of the oca2 SNP and the mc1r flanking loci in TSF2a were close to expected values (homospecific to heterospecific combinations = 17∶19 and 10∶15). Thus, the epistasis appears to be Pachón specific and to have evolved along branch C G (Fig. 1). This observation supports the hypothesis that the epistasis reflects functional interactions between oca2 and mc1r.

The putative interaction between oca2 and mc1r is particularly interesting because loss of function and hypomorphic alleles (LOF/H) at both loci have been found in several independently evolved cave populations of this species. Homozygotes for the recessive cave mc1r alleles exhibit an altered form of melanin and reduced numbers of melanophores while homozygotes for the cave alleles of oca2 lack melanin [9], [14]. Thus, oca2 is upstream of mc1r and its LOF phenotype hides the phenotypic effects of mc1r (on pigmentation). At least three cave populations (Pachón, Yerbaniz and Japonés) have LOF/H alleles at both loci. Previously, we noted these parallel instances of coevolution but had no explanation for the co-occurrences [14]. The present results suggest that if a mc1r or oca2 LOF/H becomes common in a cave population, its presence would create a positive selective pressure for an LOF/H at the other locus. That is, evolutionary change at one locus would drive change at the other.

Mc1r has more (6 edges) interactions with its neighbors than any other gene in the network. The reason for this is unclear, but mc1r is transcribed into far more mRNA species (48 presently known) than any of the other genes in the network (average±SEM = 8.3±1.2) and has been reported to be expressed in far more tissue types (190 presently known versus an average for the others of 71.1±13.3) [24]. One possibility is that the greater connectivity of mc1r in the network simply reflects its greater diversity of modes and venues of expression. Whatever the explanation, the observation suggests mc1r may have been a key gene in the evolution of cave characteristics in the *A. mexicanus* Pachón cave population, driving change at other loci in a manner similar to that suggested for oca2, above.

## Discussion

An ecological shift from a surface environment in which vision is vital to survival and where there is abundant food, to a subterranean environment in which vision is impossible and food is scarce, must create strong selection for numerous trait changes. Previous studies have shown that standing genetic variation provided much of the allelic material that was selected for in the course of cave adaptation in *Astyanax* [25] and these pre-existing variants were sited at numerous loci distributed throughout the genome [8], [9], [15]. These observations are consistent with theory suggesting that adaptation from standing variation should involve allelic substitutions at many loci [26]. With standing variation providing numerous alleles plus strong ecological pressure, adaptation to cave life may have been relatively rapid. In turn, these factors would provide the conditions for the evolution of numerous correlated (pleiotropic) effects throughout the genome, including those promoting reproductive isolation [3].

In fact, we observed extensive departures from Mendelian expectations in hybrids between surface and cave *Astyanax*. There were two main types of departures from expectations: transmission ratio distortions of blocks of linked loci, and epistatic interactions between pairs of unlinked loci. Both represent deviations from the meiotic and combinatorial norms of sexual reproduction, and are likely to reduce hybrid fitness. At present there is no evidence that the two types of departures are driven by common mechanisms, although there is no direct evidence against this possibility. The two types of deviations will be discussed separately.

### Transmission Ratio Distortion

Transmission ratio distortion occurs mainly between the diploid stage and that of fully mature sperm, as shown by the differences in allelic content of DNA from somatic tissue versus that from mature sperm. The mechanism is unknown but likely reflects genomic incompatibilities between the old and new lineage because the bias was pronounced in between-lineage hybrids but not in non-hybrids or within-lineage hybrids. We emphasize that the males used to generate the crosses in which we first detected transmission bias were not the ones tested in the DNA comparisons. Thus the effect is general to the between-lineage hybridization and not to the specific males or even the populations from which they were drawn. Furthermore, loci that exhibited significant bias in the hybrid cross tended also to exhibit bias in the DNA comparisons while those that exhibited little bias in the crosses were similarly unbiased in the DNA comparisons (Table S4; 11 of 14 vs. 0 of 3; Fisher’s exact p = 0.0294). This suggests that the DNA content biases and the transmission ratio distortion are related manifestations of the same underlying, as yet uncharacterized, phenomenon.

### Epistasis

Most of the epistasis must derive from events in the male germline because the large number of selective deaths that would be necessary for selection to occur on ova or zygotes would greatly reduce reproductive output. Mature sperm are known to contain a large and diverse pool of mRNAs and are not translationally silent [27]. Thus, in theory, the phenotype of an individual sperm could be influenced by its individual haploid genotype [28], [29]. We note that the exact patterns exhibited in Tables 1 and 2 would be generated if sperm having homospecific allelic combinations were more successful in fertilization than those with heterospecific combinations.

Recent theoretical work shows that competition among sperm from a single male can result in significant biases in genotypic content of successful sperm [29]. Essentially, in a contest between two classes of sperm, any small advantage of one over the other is magnified because of the huge number of competitors and the small number of successful outcomes.

The 22 genes examined for epistatic interactions were originally mapped as candidates for genes underlying troglomorphic trait QTL. Thus, they comprise a subset of genes that is biased towards interaction. Nevertheless, it was surprising that a network of significant interactions (Fig. 4) emerged from the analysis because the criterion used to link the nodes was non-random allelic association, one very different from traditional criteria used to construct gene networks, such as co-expression or experimental confirmation of interactions.

The network includes seven genes known or suggested to be involved with cave fish evolution: igfbp5 and tfe3, which co-map with cave trait QTL [8], [12], oca2 and mc1r, which control melanin phenotypes in cave populations [9], [14] and hsp90α, fgf8 and shh, which have variant patterns of expression in cave embryos that influence the development of cave phenotypes [21]–[23]. Because the criterion for including genes in this network is unique, their inclusion is independent confirmation of their potential importance in the cave adaptation of the Pachón population. The analysis of co-segregation patterns from hybrid genomes may generally be useful in detecting coadapted gene complexes underlying evolutionary phenotypic change, providing a novel way to illuminate the evolutionary history of lineages.

Our working hypothesis is that the biases against HET combinations reflect functional incompatibilities of alleles from the two different populations and that these incompatibilities have deleterious effects on sperm as well as zygote function. This hypothesis concerning mechanism is speculative, but it is clear that the epistatic interactions could act as post-mating hybridization barriers. Thus, interacting loci may be good candidates for “speciation genes,” with interacting cave specific alleles fixed during adaptation to cave life. Our observations imply strong support for the by-product hypothesis of ecological speciation [1], [2].

## Materials and Methods

Ethics Statement: All animal work was conducted according to relevant national and international guidelines. This work was approved by the University Animal Welfare Committee of New York University (Protocol numbers 00–1022 and 00–1015).

## Supporting Information

Figure S1 **Linkage maps for the PSF2 cross.** Markers exhibiting segregation distortion are indicated in red for preferential transmission of the surface alleles or blue for preferential transmission of the Pachón cave alleles.
(PDF)

Figure S2 **Linkage maps for the PSBC cross.** Markers exhibiting segregation distortion are indicated in red for preferential transmission of the surface alleles or blue for preferential transmission of the Pachón cave alleles.
(PDF)

Figure S3 **Linkage maps for the MSBC cross.** Markers exhibiting segregation distortion are indicated in red for preferential transmission of the surface alleles or blue for preferential transmission of the Molino cave alleles.
(PDF)

Figure S4 **Linkage maps for the TSF2a cross.** Markers exhibiting segregation distortion are indicated in red for preferential transmission of the surface alleles or blue for preferential transmission of the Tinaja cave alleles.
(PDF)

Figure S5 **Linkage maps for the TSF2b cross.** Markers exhibiting segregation distortion are indicated in red for preferential transmission of the surface alleles or blue for preferential transmission of the Tinaja cave alleles.
(PDF)

Table S1 **Summary statistics for the five crosses analyzed: Pachon F_2_ (PSF2), Tinaja F_2_a (TSF2a), Tinaja F_2_b (TSF2b), Pachón BC (PSBC) and Molino BC (MSBC).** Marker codes: microsatellites = a; SNPs = b; genes = c.
(DOCX)

Table S2 **Spreadsheet with raw data for the PSF2 cross giving genotypic counts at scored loci.** Loci are ranked in descending order of significant bias, based on calculated χ2 value (df = 1). Linkage data is given for the 33 loci nominally significant at the P = 0.001 level. The 14 unlinked loci used to calculate the severity of zygotic selection required to produce the observed bias are flagged in column AA>
(XLSX)

Table S3 **Homospecific combinations (HOM) of alleles between pairs of unlinked loci outnumber heterospecific combinations (HET) at all levels of increasing departure from equality as measured by Χ^2^ value.**
(DOCX)
