# Supplementary figures and images for: Genomic Consequences of Ecological Speciation in Astyanax Cavefish
Source: PLoS One. 2013 Nov 19;8(11):e79903. doi: 10.1371/journal.pone.0079903 (PMC3833966; doi:10.1371/journal.pone.0079903)

1

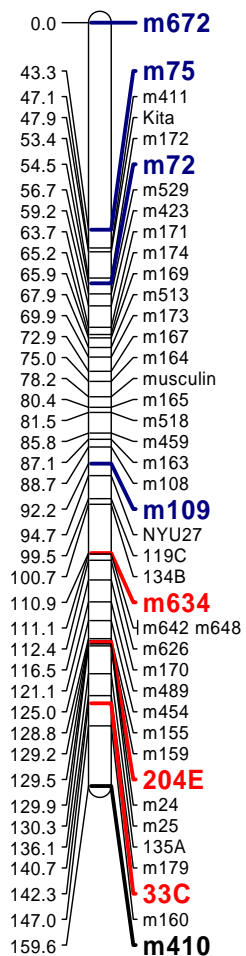

2A

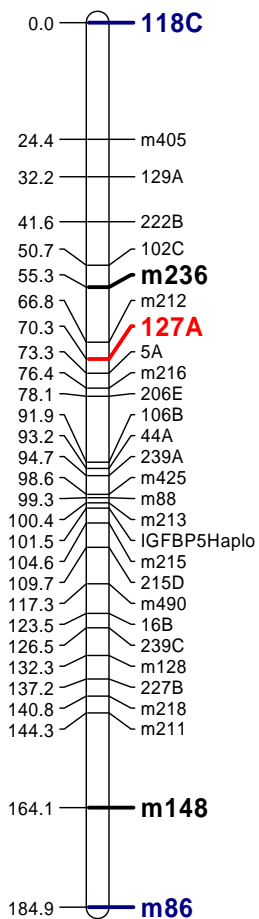

2B

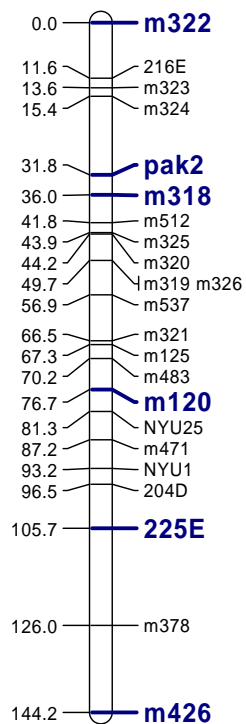

3A

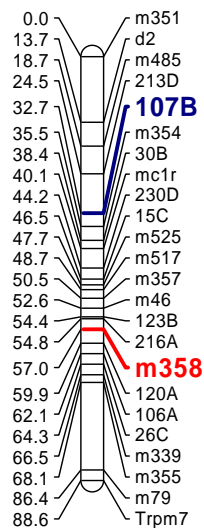

3B

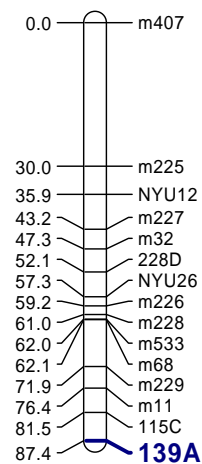

4

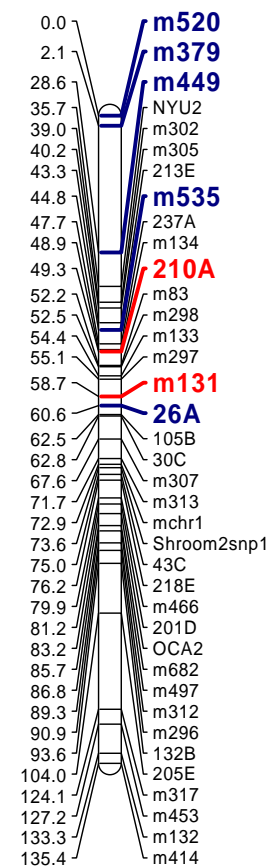

5

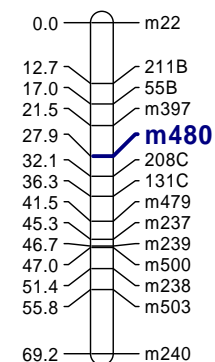

6

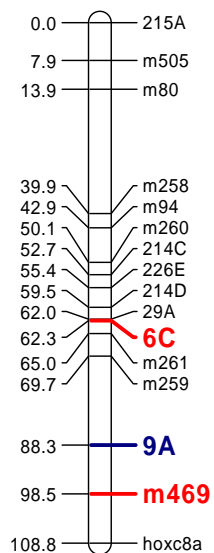

7

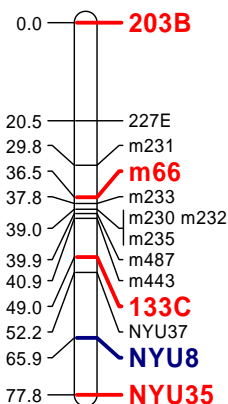

8

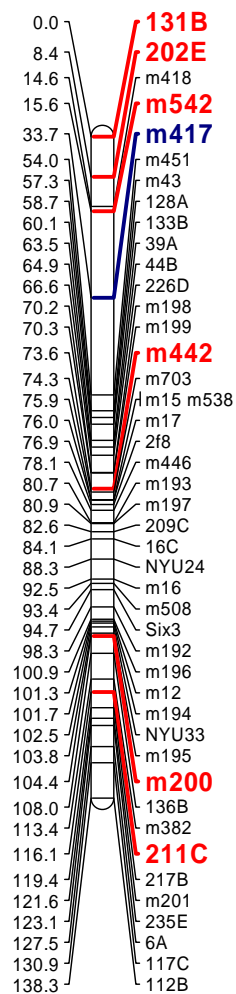

9

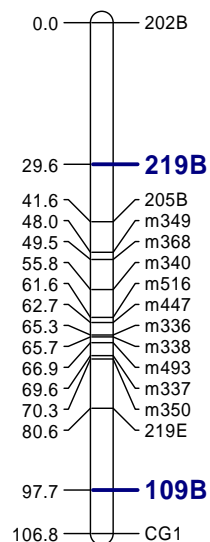

10

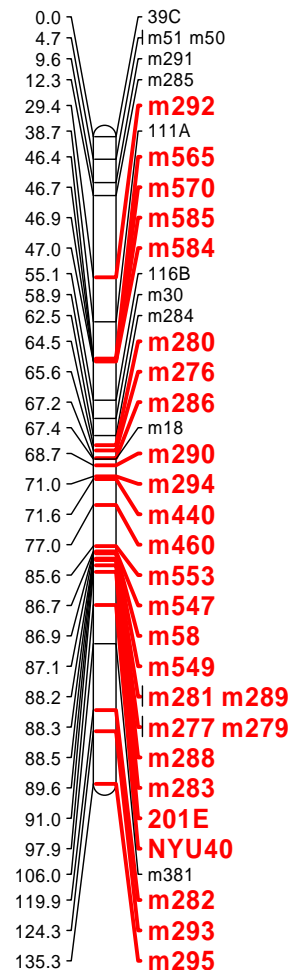

11

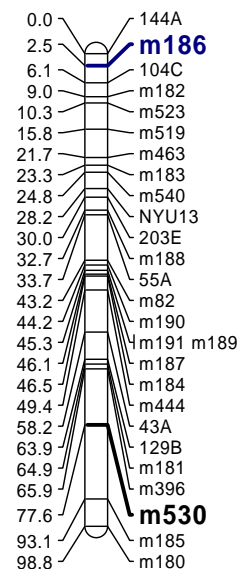

12

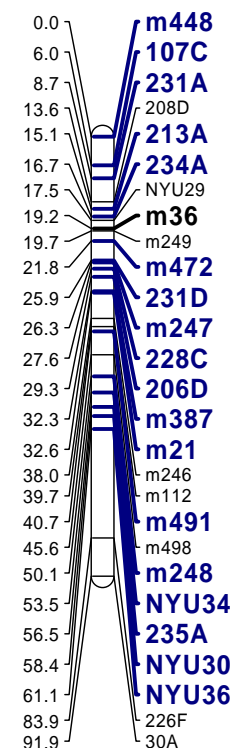

13

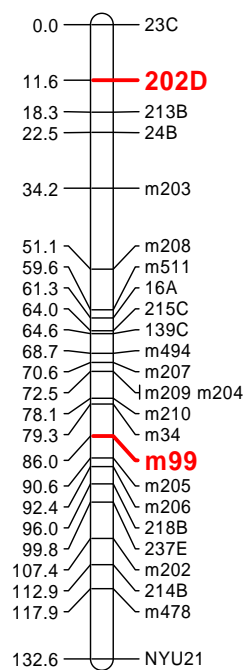

14

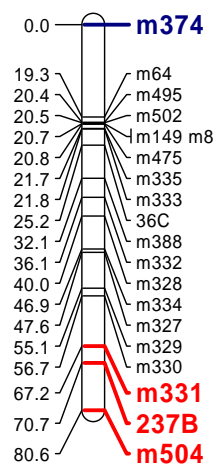

15

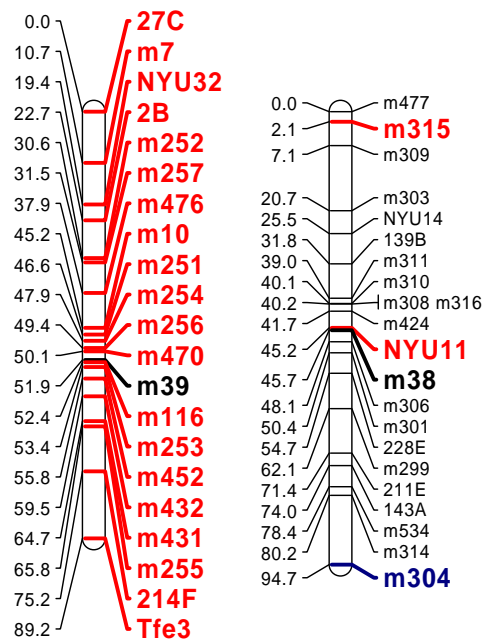

16

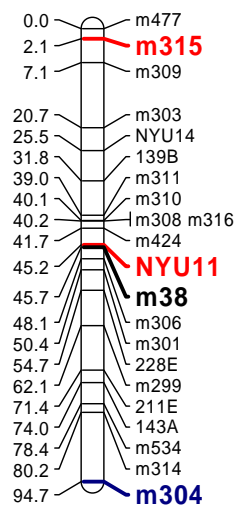

17

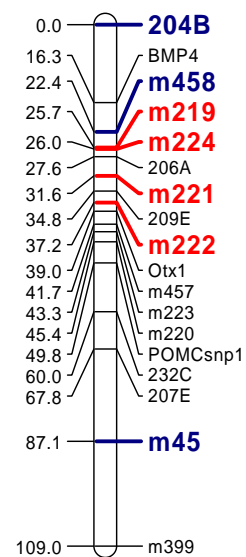

18

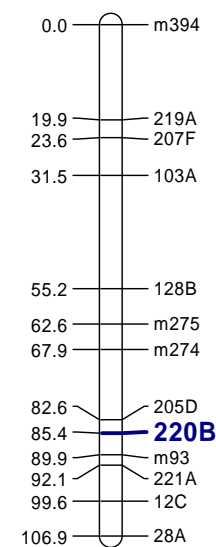

19

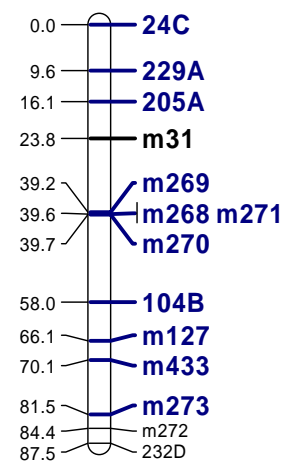

20

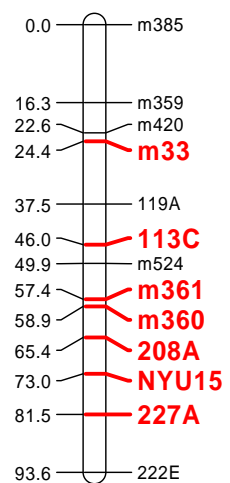

21

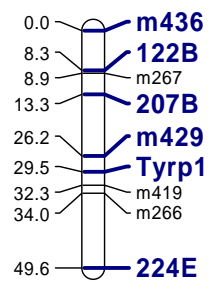

23

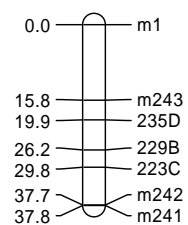

24

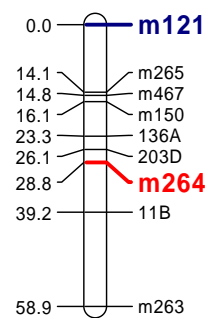

25

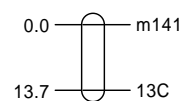

Supplement: Figure S1 — Linkage maps for the PSF2 cross. Markers exhibiting segregation distortion are indicated in red for preferential transmission of the surface alleles or blue for preferential transmission of the Pachón cave alleles. (PDF) [file pone.0079903.s001.pdf]

1

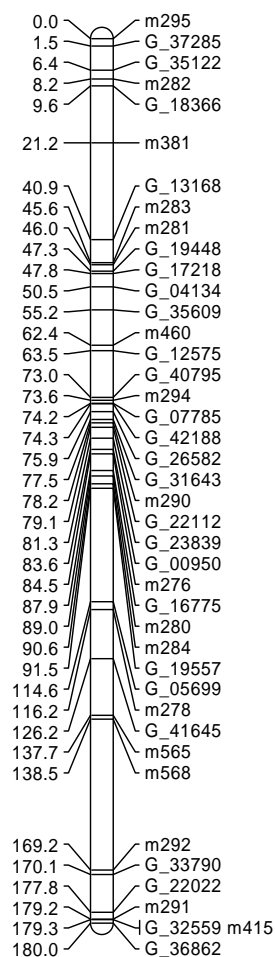

2

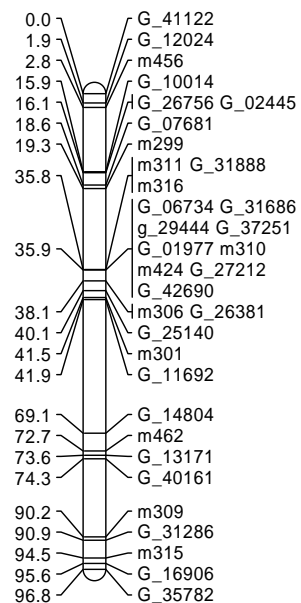

3

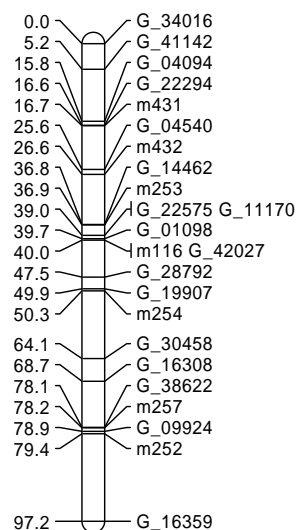

4

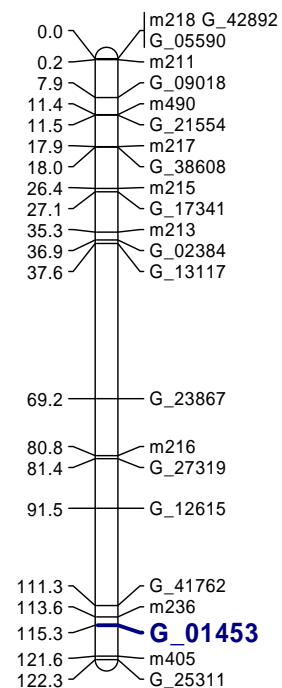

5

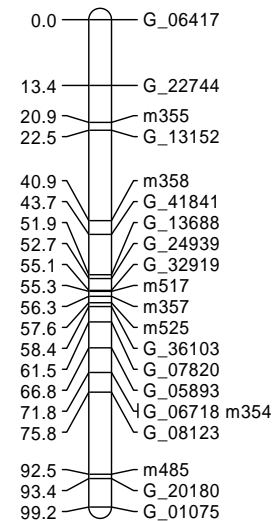

6

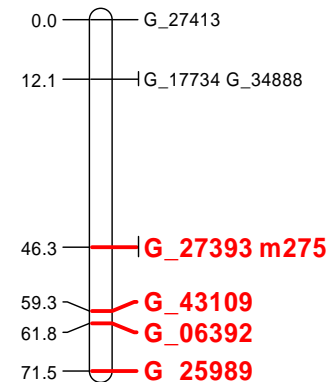

7

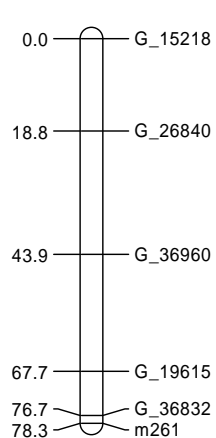

8

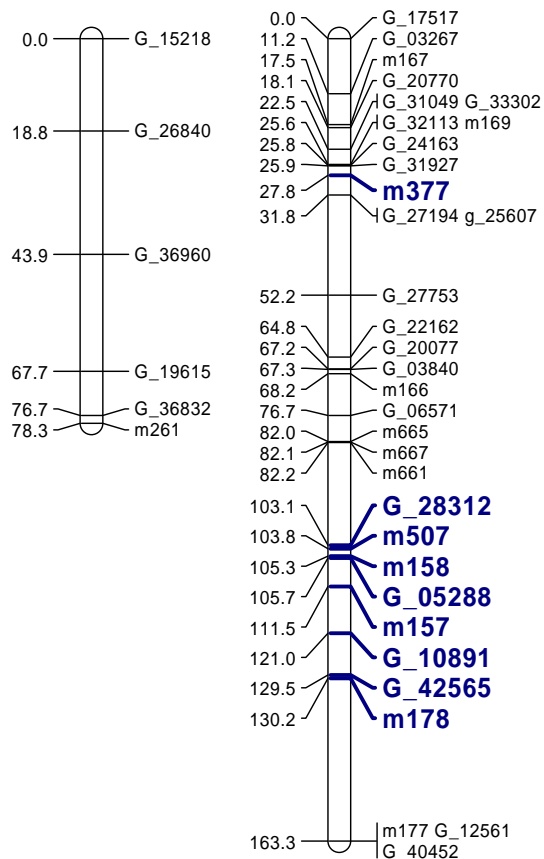

9

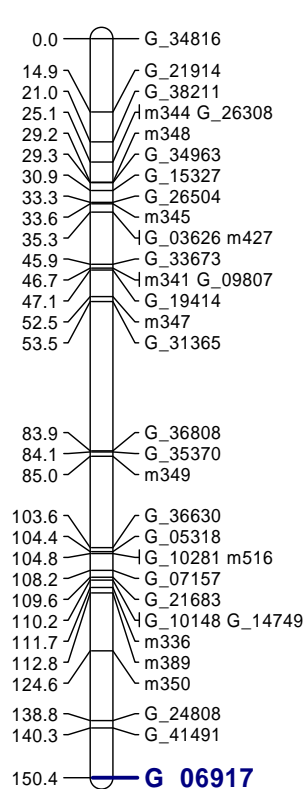

10

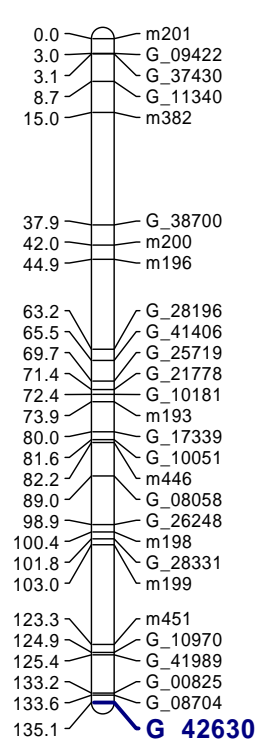

11

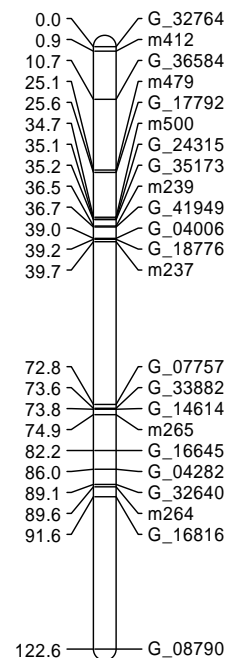

12

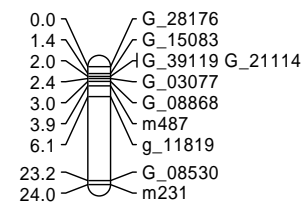

13

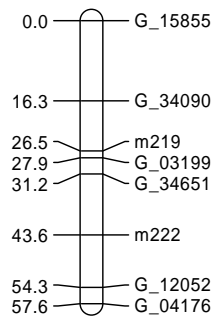

14

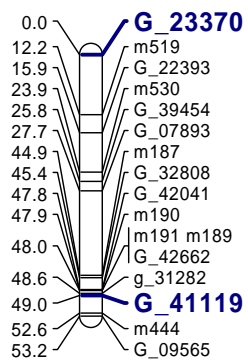

15

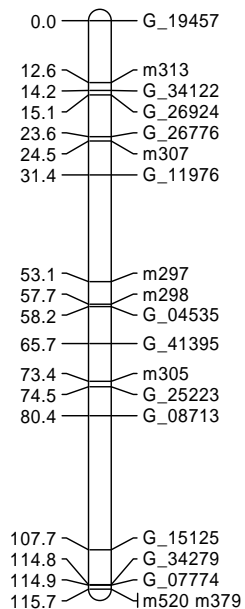

16

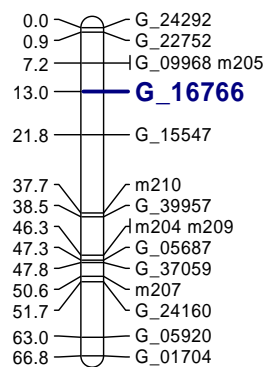

17

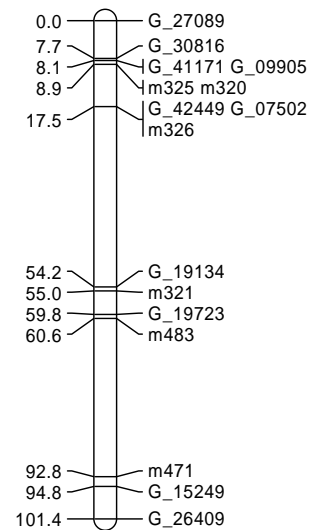

18

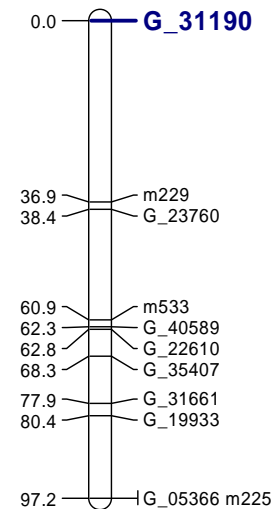

19

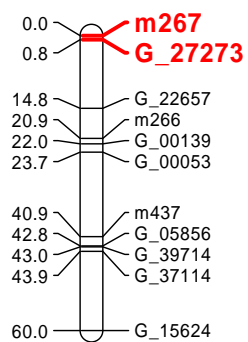

20

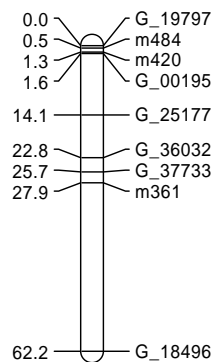

21

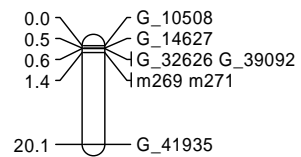

22

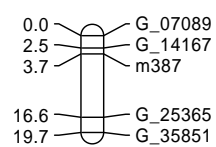

23

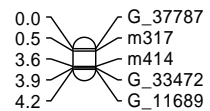

24

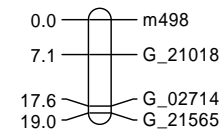

25

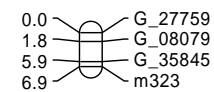

26

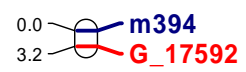

27

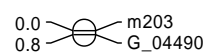

28

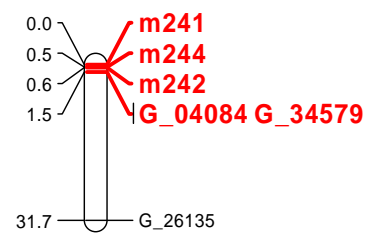

29

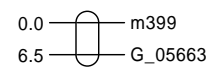

Supplement: Figure S2 — Linkage maps for the PSBC cross. Markers exhibiting segregation distortion are indicated in red for preferential transmission of the surface alleles or blue for preferential transmission of the Pachón cave alleles. (PDF) [file pone.0079903.s002.pdf]

1

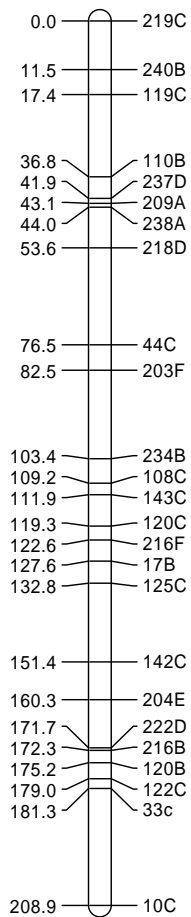

2

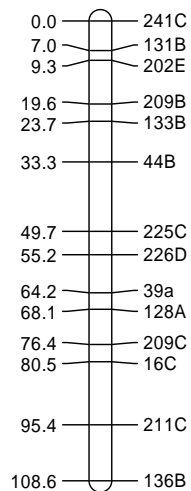

3

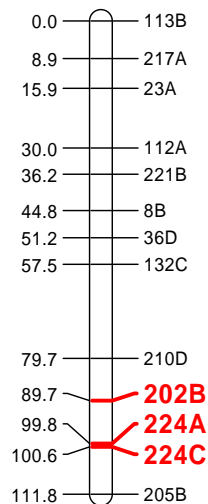

4

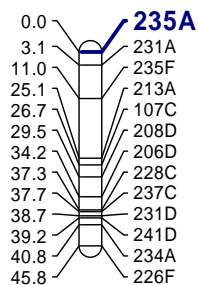

5

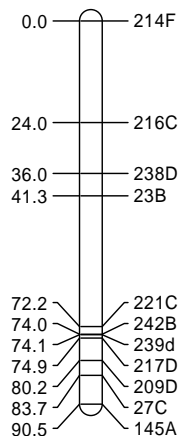

6

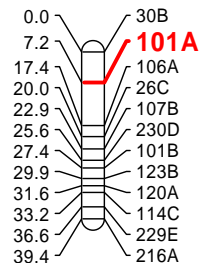

7

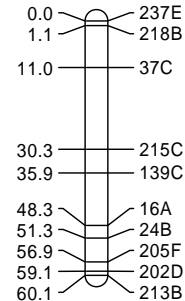

8

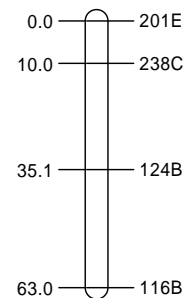

9

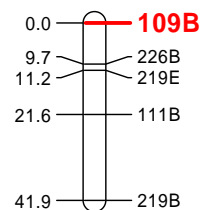

10

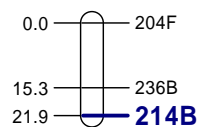

11

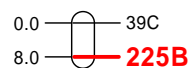

12

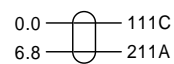

13

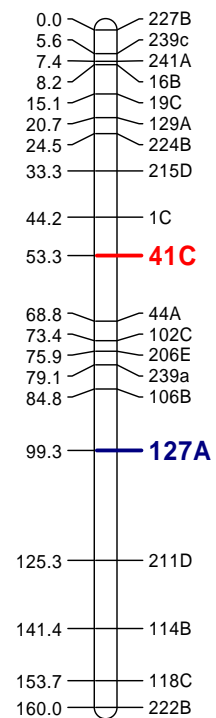

14

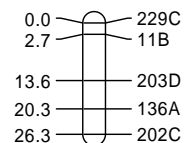

15

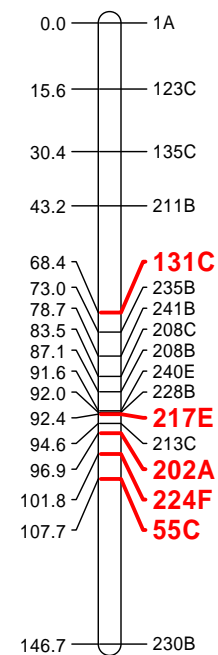

16

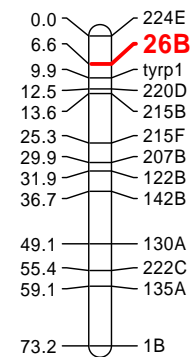

17

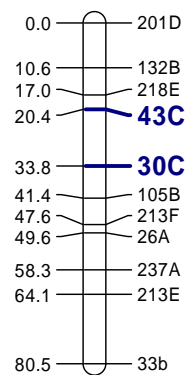

18

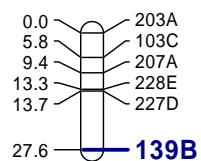

19

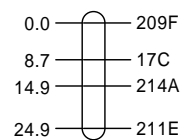

20

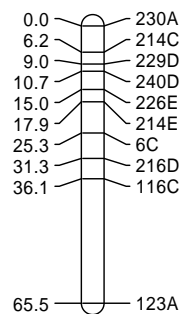

21

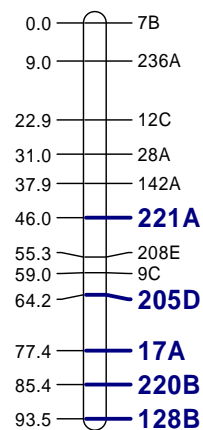

22

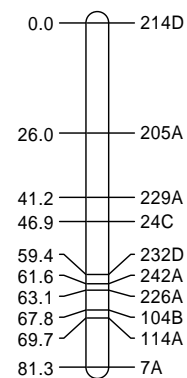

23

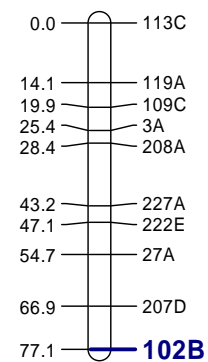

24

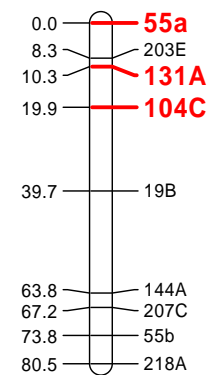

25

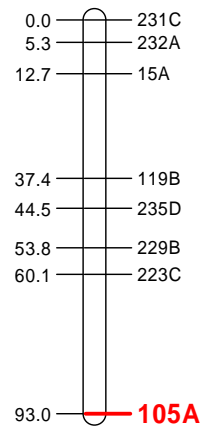

26

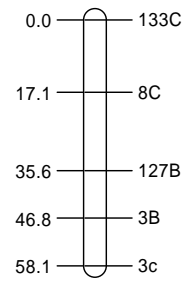

27

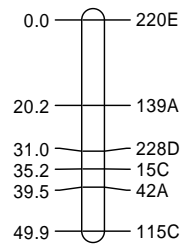

28

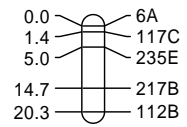

29

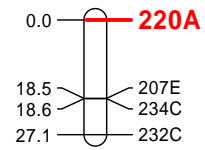

30

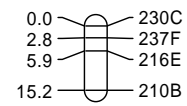

31

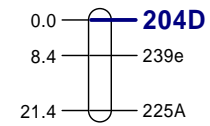

32

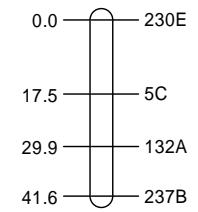

33

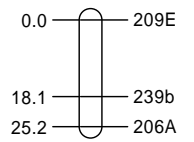

**34**

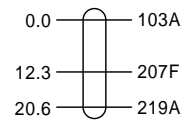

**35**

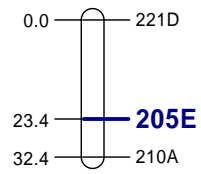

**36**

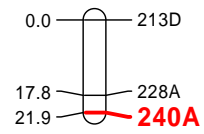

**37**

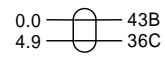

**38**

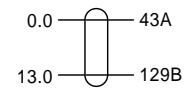

Supplement: Figure S3 — Linkage maps for the MSBC cross. Markers exhibiting segregation distortion are indicated in red for preferential transmission of the surface alleles or blue for preferential transmission of the Molino cave alleles. (PDF) [file pone.0079903.s003.pdf]

1A

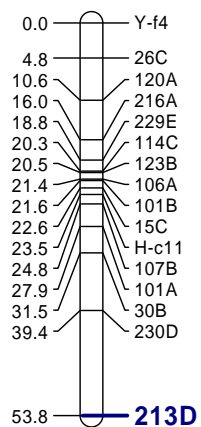

1B

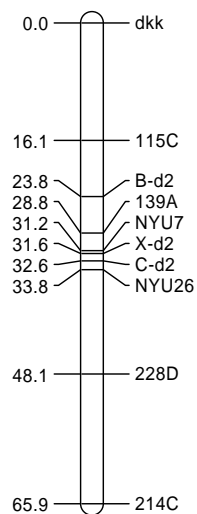

2

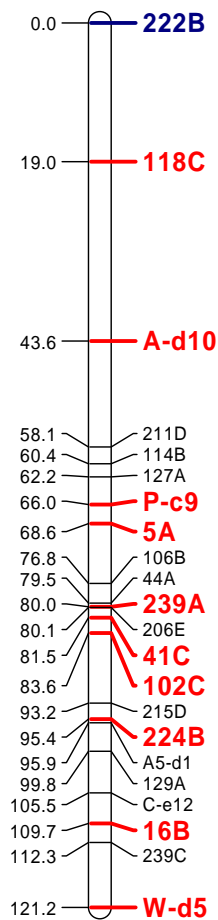

3

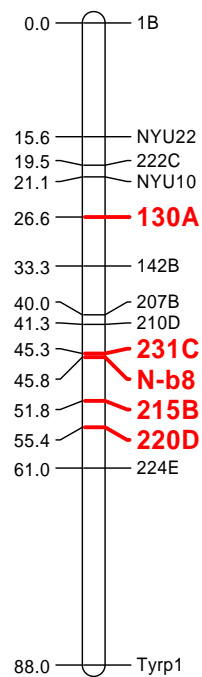

4

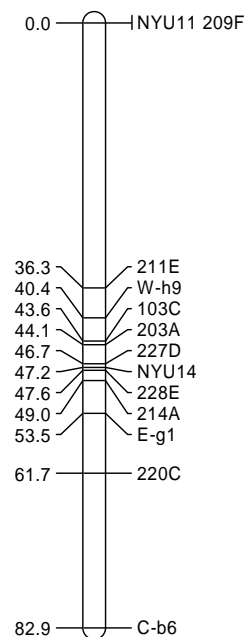

5

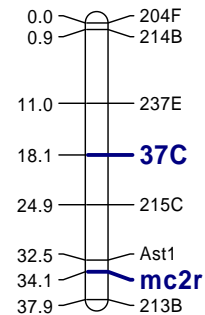

6

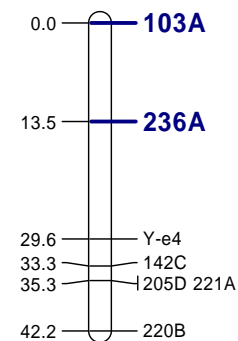

7

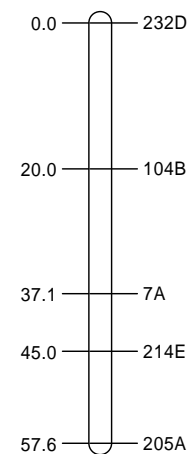

8

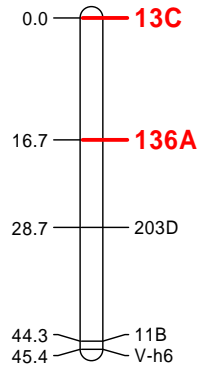

9

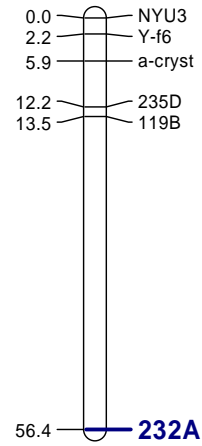

10

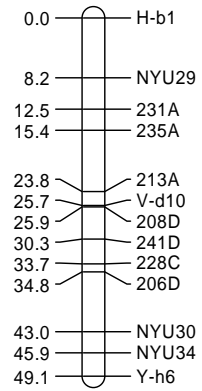

11A

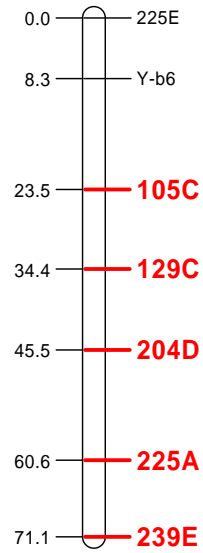

11B

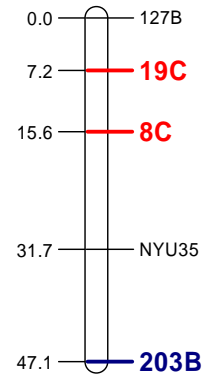

12

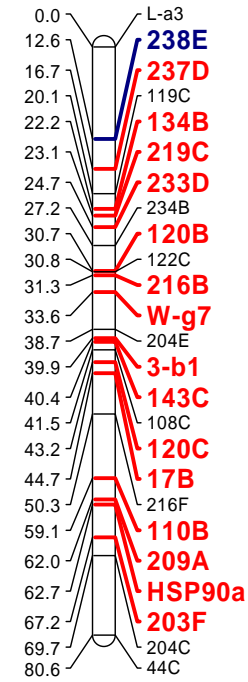

13

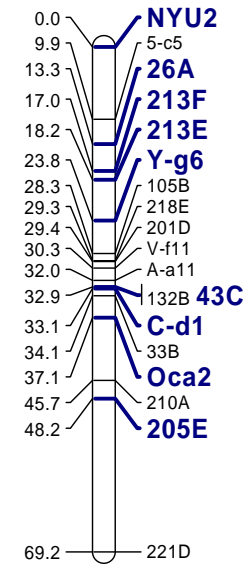

14

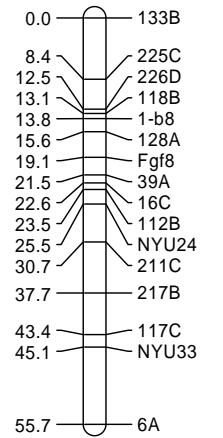

15

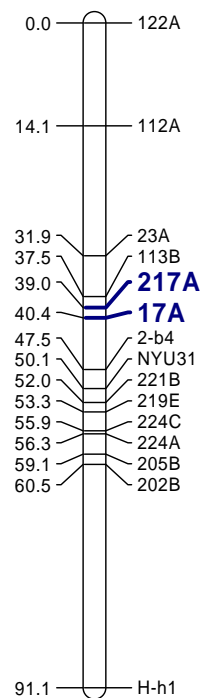

16

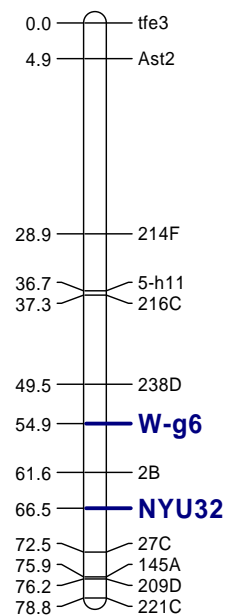

17

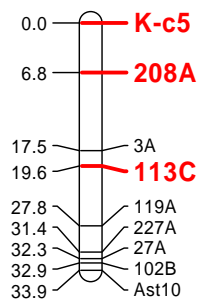

18

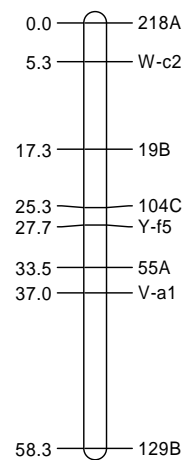

19

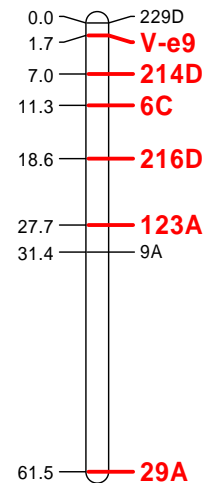

20

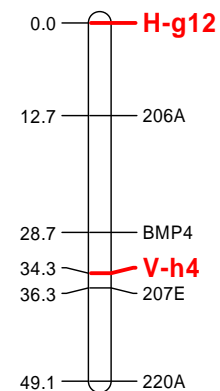

21

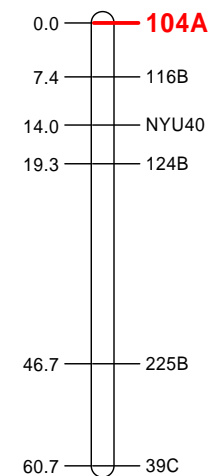

22

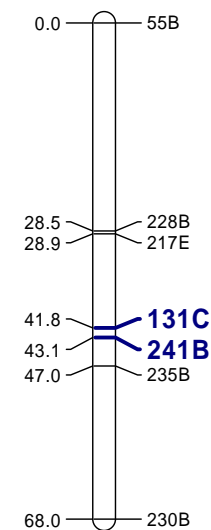

23

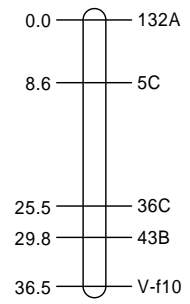

Supplement: Figure S4 — Linkage maps for the TSF2a cross. Markers exhibiting segregation distortion are indicated in red for preferential transmission of the surface alleles or blue for preferential transmission of the Tinaja cave alleles. (PDF) [file pone.0079903.s004.pdf]

1

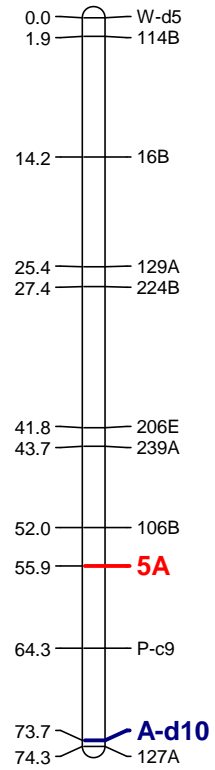

2

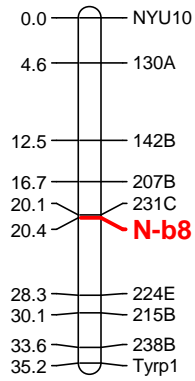

3

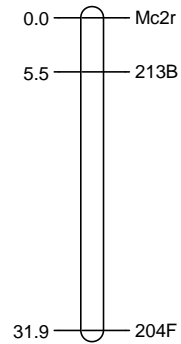

4

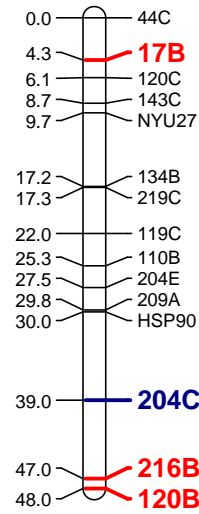

5

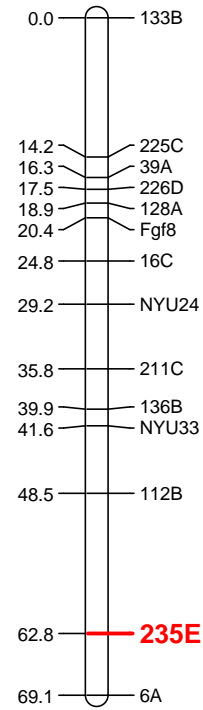

6

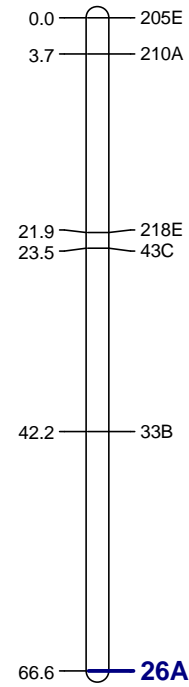

7

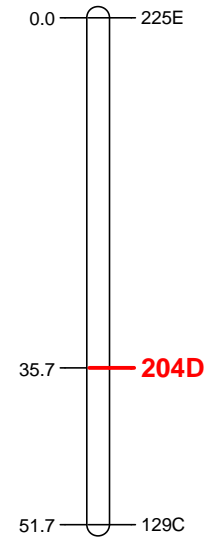

8

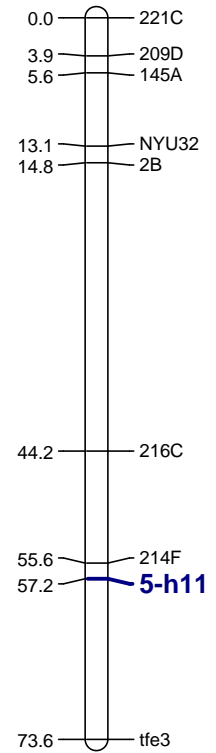

9

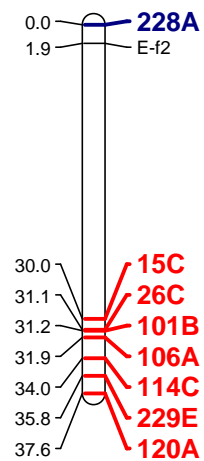

10

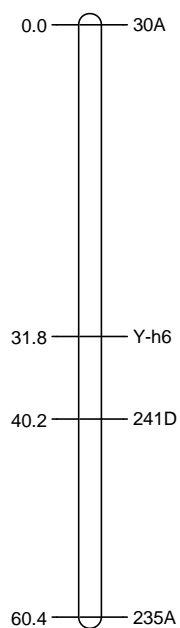

11

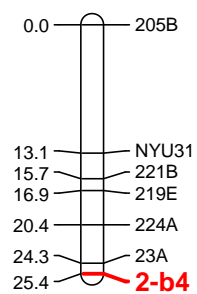

12

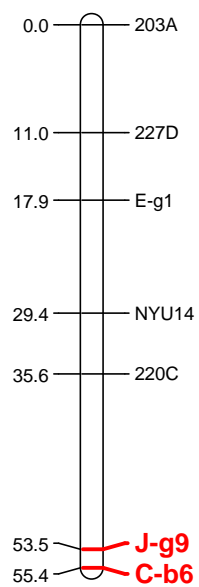

13

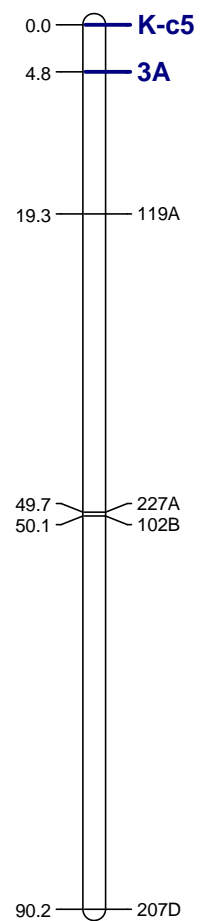

14

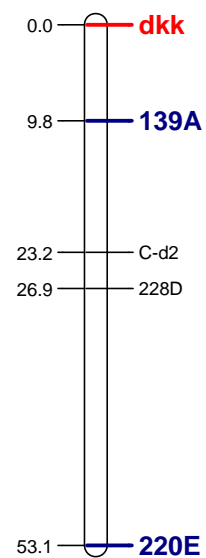

15

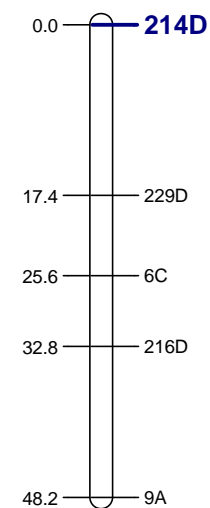

16

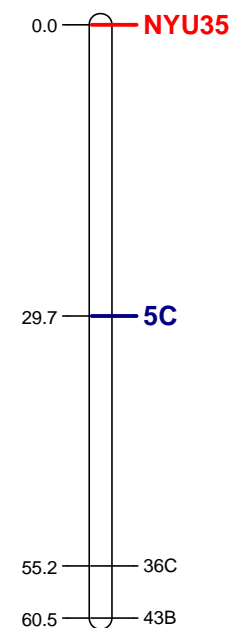

17

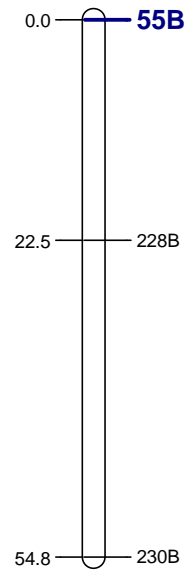

18

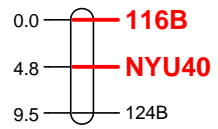

19

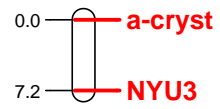

20

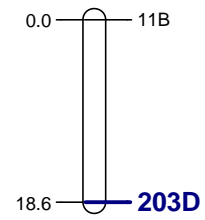

21

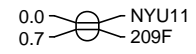

Supplement: Figure S5 — Linkage maps for the TSF2b cross. Markers exhibiting segregation distortion are indicated in red for preferential transmission of the surface alleles or blue for preferential transmission of the Tinaja cave alleles. (PDF) [file pone.0079903.s005.pdf]
