# Supplementary material for: Genomic Consequences of Ecological Speciation in Astyanax Cavefish
Source: PLoS One. 2013 Nov 19;8(11):e79903. doi: 10.1371/journal.pone.0079903 (PMC3833966; doi:10.1371/journal.pone.0079903)
Supplement: Table S1 — Summary statistics for the five crosses analyzed: Pachon F2 (PSF2), Tinaja F2a (TSF2a), Tinaja F2b (TSF2b), Pachón BC (PSBC) and Molino BC (MSBC). Marker codes: microsatellites = a; SNPs = b; genes = c. (DOCX) [file pone.0079903.s006.docx]

Table S1. Summary statistics for the five crosses analyzed: Pachon F_2_ (PSF2), Tinaja F_2_a (TSF2a), Tinaja F_2_b (TSF2b), Pachón BC (PSBC) and Molino BC (MSBC). Marker codes: microsatellites = a; SNPs = b; genes = c.

|  | PSF2 | TSF2a | TSF2b | PSBC | MSBC |
| --- | --- | --- | --- | --- | --- |
| # Loci | 541 | 268 | 131 | 431 | 296 |
| # Individuals | 539 | 288 | 192 | 127 | 119 |
| # LG | 26 | 24 | 21 | 29 | 38 |
| Map Length (cM) | 2584 | 1563 | 972 | 2065 | 2189 |
| Resolution cM | 5 | 6.4 | 8.8 | 5.1 | 8.5 |
| Markers | a, b | a, c | a | b | a |
| # Biased Regions | 18 | 18 | 9 | 3 | 5 |
| Bias Extent (cM) | 382 | 181 | 45 | 55 | 74 |
| % Genome | 14.8 | 11.6 | 4.6 | 2.6 | 3.4 |
| LG with bias | 11 | 12 | 8 | 3 | 5 |
