# Supplementary material for: Genomic Consequences of Ecological Speciation in Astyanax Cavefish
Source: PLoS One. 2013 Nov 19;8(11):e79903. doi: 10.1371/journal.pone.0079903 (PMC3833966; doi:10.1371/journal.pone.0079903)
Supplement: Table S3 — Homospecific combinations (HOM) of alleles between pairs of unlinked loci outnumber heterospecific combinations (HET) at all levels of increasing departure from equality as measured by Χ2 value. (DOCX) [file pone.0079903.s008.docx]

Table S3. Homospecific combinations (HOM) of alleles between pairs of unlinked loci outnumber heterospecific combinations (HET) at all levels of increasing departure from equality as measured by Χ^2^ value.

| χ^2^ Bin | HET |  | HOM |
| --- | --- | --- | --- |
| 0-1 | 33489 |  | 34551 |
| 1-2 | 5483 |  | 7260 |
| 2-3 | 2573 |  | 3512 |
| 3-4 | 1109 |  | 1772 |
| 4-5 | 603 |  | 1024 |
| 5-6 | 343 |  | 567 |
| 6-7 | 222 |  | 340 |
| 7-8 | 109 |  | 158 |
| 8-9 | 65 |  | 96 |
| 9-10 | 34 |  | 56 |
| 10-11 | 18 |  | 29 |
| 11-12 | 11 |  | 19 |
| 12-13 | 4 |  | 8 |
| 13+ | 5 |  | 12 |
| TOTALS | 44068 |  | 49405 |
| GRAND TOTAL = 93473 |  |  |  |
